# Bilateral testicular metastases of medullary thyroid carcinoma in an adult male with multiple endocrine neoplasia 2A syndrome: case report and review of literature

**DOI:** 10.1530/ETJ-21-0016

**Published:** 2022-02-16

**Authors:** Francesca Orsolini, Alessandro Prete, Pierpaolo Falcetta, Domenico Canale, Fulvio Basolo, Greta Alì, Francesca Manassero, Paolo Vitti, Rossella Elisei, Eleonora Molinaro

**Affiliations:** 1 Department of Clinical and Experimental Medicine, Endocrine Unit, University of Pisa, Pisa, Italy; 2 Department of Surgical, Medical and Molecular Pathology and Critical Care Medicine, University of Pisa, Pisa, Italy; 3 Division of Urology, Department of Translational Research and of New Surgical and Medical Technologies, University of Pisa, Pisa, Italy

**Keywords:** testicular metastases, medullary thyroid cancer, multiple endocrine neoplasia 2A syndrome

## Abstract

**Introduction:**

Medullary thyroid cancer (MTC) is a rare endocrine tumor, which can be sporadic or familial, as a component of multiple endocrine neoplasia 2 (MEN2). Overall, 10% of MTC cases have already developed at presentation or will develop metastasis during follow-up. Testicular metastases are exceptional and only one case of unilateral testis involvement by metastatic MTC has been already reported in literature. We described the first known case of asymptomatic bilateral testicular MTC metastases, discovered incidentally at testicular ultrasound (US) performed for unrelated reasons.

**Case presentation:**

A Latin American 32-year-old man with MEN 2A syndrome and metastatic MTC underwent andrological and urological examination due to premature ejaculation. US imaging showed two symmetrical hypoechoic lesions involving both testes. Suspecting a bilateral testicular cancer, the patient underwent excision biopsy of both testicular lesions. Histopathology and immunohistochemical examinations documented metastatic MTC of both testicular lesions.

**Conclusion:**

Beyond its rarity, testis should be considered as a potential metastatic site of MTC, especially in patients with advanced disease.

**Established facts:**

- Distant metastases are present at the diagnosis in 10–15% of patients with medullary thyroid carcinoma (MTC).
- Testicular metastases are anecdotal. Only one case of unilateral testis involvement by metastatic MTC has been reported in the literature.

**Novel insights:**

- Testis should be considered as a possible site of metastases in patients with diffuse metastatic MTC.
- Testicular ultrasound could be considered as an useful tool for the evaluation and follow-up of metastatic MTC.

## Introduction

Multiple endocrine neoplasia 2A (MEN2A) syndrome is an autosomal dominant disorder that includes medullary thyroid carcinoma (MTC), pheochromocytoma and hyperparathyroidism. MTC is the most common manifestation (90–100%) of MEN2A and often represents the first clinical presentation. Pheochromocytoma and hyperparathyroidism develop in approximately 50% and 25% of individuals, respectively (1). The molecular basis for familial MTC is usually represented by a dominant activating mutation of the REarranged during Transfection (*RET*) proto-oncogene, mostly located in the extracellular, cysteine-rich region of exon 10 (including codons 609, 611, 618 and 620) and exon 11 (including codons 630 and 634) of the gene (1, 2, 3, 4).

In 30–50% of the cases, MTC is accompanied by metastases in cervical and/or paratracheal lymph nodes, and the tumor spreads also to the upper and anterior mediastinal lymph nodes. Distant metastases are present at the diagnosis in 10–15% of patients (2, 3, 5). Other than to the soft tissues of the neck region, MTC spreads first to the liver (45%) and bone (45%) and less commonly to lung (33%), brain (1–5%) or skin (few cases reported) (6). Metastases to adrenal glands, pleura, heart, ovary, pancreas, pituitary, eye and breast have been rarely/exceptionally reported (7, 8, 9, 10, 11, 12, 13, 14, 15). To our knowledge, a single case of unilateral testicular metastasis of sporadic MTC was reported before by Appetecchia *et al.* (16).

Serum tumoral markers of metastatic MTC are both calcitonin (CTN) and carcinoembryonic antigen (CEA). Their levels correlate with the tumor burden, and their doubling time is associated with the outcome of the disease (17).

In this article, we report the unusual case of a patient with MEN2A syndrome with bilateral testicular metastases from metastatic and advanced MTC, incidentally discovered at ultrasound (US) imaging and histologically confirmed.

## Case presentation

The case was a Latin American 32-year-old man affected by MEN2A syndrome with a germline *RET*Cys618Arg mutation localized in exon 10 and characterized by MTC with cervical and mediastinal lymph node metastases and bilateral pheochromocytoma.

In 2010, at age of 24 years, due to instrumental detection of an adrenal incidentaloma along with elevated metanephrines, the patient underwent right adrenalectomy for pheochromocytoma in another country. Five years later, a diagnosis of MTC with cervical and mediastinal lymph node metastases was performed, and the patient underwent total thyroidectomy, central and lateral lymph node dissection and external beam radiotherapy. Unfortunately, CTN and CEA levels before surgery are not available, but we found that CTN was >2000 ng/L at first postoperative evaluation in 2015. Later, in 2017, he moved to Italy and he was initially referred to another hospital. In April 2017, CTN and CEA were 16,527 pg/mL and 118.7 ng/mL, respectively. Contrast-enhanced CT showed the presence of cervical and mediastinal lymph node metastases and a single lung micronodule of uncertain significance. Biochemical and imaging evaluation documented in addition a pheochromocytoma in the left adrenal gland, so the patient underwent left adrenalectomy. In agreement with a family history that showed that patient’s father died (at age of 56 years) of thyroid carcinoma (as well as paternal grandfather and uncle) and with clinical course, genetic analysis was performed and revealed a *RET*Cys618Arg heterozygous germline mutation. Genetic analysis was negative in his surviving relatives (sister and mother).

He was referred to our department in April 2018 after the appearance of cervical and mediastinal lymph nodes metastases and lung micronodules at CT scan combined with a significant increase in both CTN and CEA serum tumor markers.

**Figure 1:**
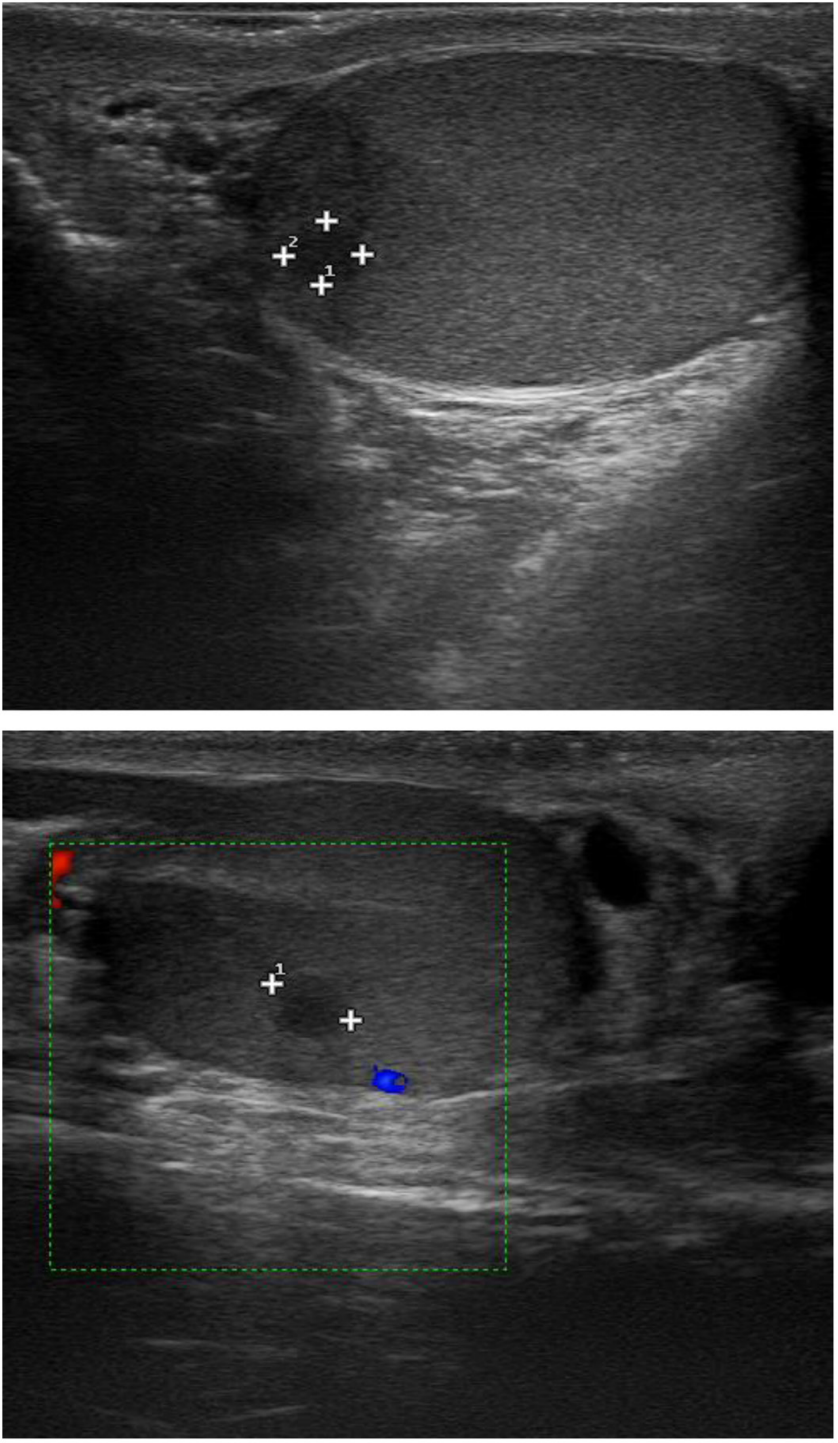
Ultrasound imaging. Hypoechoic lesion of 5 mm in the middle part of the right testis, without a clear vascular pattern.

**Figure 2:**
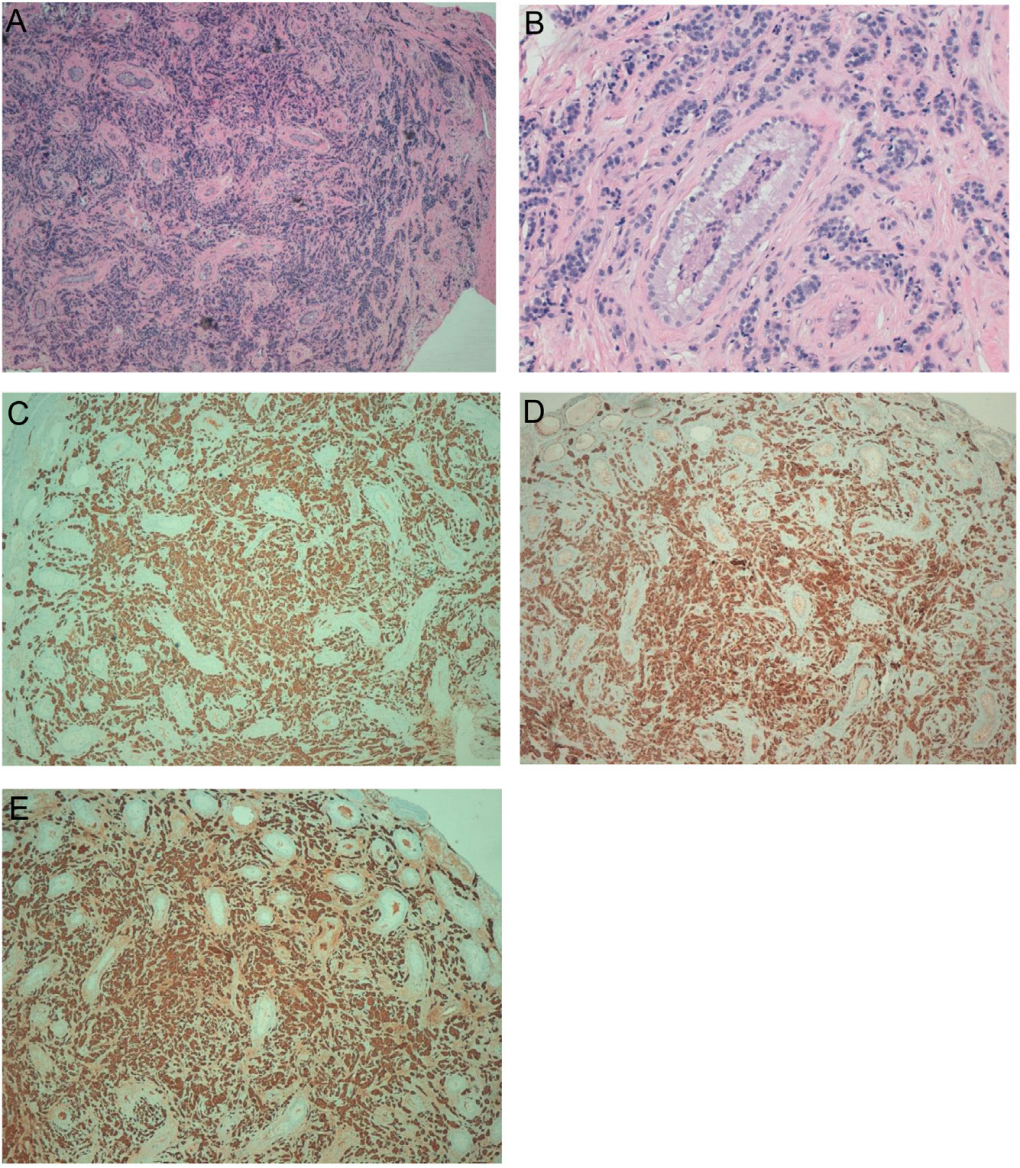
Immunohistochemical examinations. Hematoxylin–eosin coloring (A and B). Tumor cells show positivity for CTN (C), CD56 (D) chromogranin A (E).

At first evaluation in our institute, serum CTN was 18,889 ng/L (n.v. < 11.5) and CEA was 167.9 µg/L (n.v. < 5.2) (Table 1). Urinary-fractionated metanephrines were in the normal range. CT scan confirmed the presence of multiple vascular-rich lymph nodes in the cervical region and upper mediastinum and lung-scattered micronodules. The patient was asymptomatic, except for an apparently unrelated premature ejaculation. For this symptom, he underwent andrological and urological examination. No palpable nodule in the testes was found, but US imaging showed two symmetrical hypoechoic lesions of 5 mm in the lower part of the left testis and in the middle part of the right testis, without a clear vascular pattern (Fig. 1). Suspecting a testicular cancer, according to the most recent guidelines (18), beta-human chorionic gonadotropin (BHCG), alpha-fetoprotein (AFP) and lactate dehydrogenase (LDH) were evaluated and were within normal values (BHCG < 0.5 UI/L, n.v. < 1 UI/L, AFP 1.6 µg/L, n.v. < 7 µg/L, LDH 171 U/L, n.v. 135–225 U/L). Therefore, the patient underwent enucleoresection of both testicular lesions. Histopathology revealed the presence of atypical cells with focal granular cytoplasm and ‘salt and pepper’ chromatin, morphologically resembling MTC. Immunohistochemistry showed reactivity for chromogranin A, CTN, CEA, CD56 (neural cell adhesion molecule), transcriptional thyroid factor 1, synaptophysin, OCT4 (cytoplasmatic pattern), CK-Pan (‘dot-like’ pattern), thus confirming the diagnosis of metastases from MTC (Fig. 2). No reactivity was shown for anti-placental alkaline phosphatase, CD30, CD117, inhibin, α-FP. The MIB-1 proliferation index was about 10%. The evaluation of serum tumor markers in the postoperative period showed a transient reduction of their values, mainly of CTN (Table 1).

**Table 1:** Medullary thyroid markers before and after enucleoresection of the testicular lesions.

Markers	Before surgery	4 days after surgery	2 months after surgery	Normal values
CTN (ng/L)	18,889	9045	18,024	<11.5
CEA (µg/L)	167.9	144.1	161.8	<5.2

## Discussion

Testicular metastases from solid tumors are extremely rare; it is difficult to establish their real frequency, as in the majority of cases they are detected incidentally at autopsy or during therapeutic orchiectomy for prostatic cancer (19). The incidence of testicular metastases in autopsy studies of subjects with solid tumors ranges from 0.59% to 2.5% (19, 20, 21). The most common neoplasia to metastasize to the testis is prostatic carcinoma (29–35%), followed by lung (16%), kidney (9%), colon (7%) and stomach tumors (only approximately 14 cases reported worldwide) (21, 22). Rare cases of metastases from retinoblastoma, appendix and small bowel carcinoid, rectum and pancreas have been also reported, such as the involvement of the testis by primary tumors in the contralateral testis (20, 23, 24). There are few cases of testicular metastases from primary neuroendocrine tumors (25, 26, 27). Secondaries to the testes are most often unilateral. Bilateral metastases are described in about 15–20% of cases from prostate cancer, colorectal cancer, lung adenocarcinoma and renal cell carcinoma, and they are commonly regarded as a sign of advanced disease and accompanied by cancer spreading to other sites (24, 28, 29, 30).

Unilateral testicular metastases from MTC have been previously reported only in one case by Appetecchia *et al.* (16), who described a 73-year-old Caucasian man presenting with sporadic metastatic MTC and a painless nodule in the right testis associated with a palpable inguinal lymph node, histologically defined as testicular and inguinal lymph node metastases after orchiectomy and excision biopsy.

In the present case, we described a young man with syndromic advanced MTC with metastases involving both testes. It is known that testicular metastases are commonly present in widespread tumors (19, 31). Indeed, also in our case, the elevated basal levels of CTN (18,889 ng/L) and CEA (167.9 µg/L) were indicative of an extensive tumor burden, and CT scan showed lymph node involvement and distant metastases with diffused lung micronodules. In contrast to the case presented by Appetecchia *et al.*, the patient reported no symptoms or signs related to a testicular mass, other than premature ejaculation unlikely related to the testicular lesions (32). After the enucleoresection, the patient experienced a decrease in both CTN and CEA levels (CTN 18,889 ng/L and CEA 167.9 µg/L before surgery vs CTN 9045 ng/L and CEA 144.1 µg/L 4 days after surgery); however, this decrease was only brief and transient, and, 2 months after surgery, CTN and CEA reached pre-surgical levels. We cannot explain this phenomenon, but it is conceivable that the resection of these small testicular metastatic lesions could not have a big impact on the serum levels of the tumors markers. Indeed, the CEA that is more related to the tumor burden was only slightly reduced. As far as the secretion of CTN is concerned, we can only suppose an impact of the operative distress on the CTN secretion since it decreased immediately after the intervention but then it grew back without being accompanied by an evident increase in the tumor mass at CT scan.

More than 40% of testicular metastases are asymptomatic (33, 34), and most of them are found incidentally at orchiectomy for prostatic carcinoma or at autopsy. In several cases, testicular metastases unnoticed with usual imaging procedures such as CT scan or MRI are detected using different diagnostic tools (25, 26, 27, 33, 35). In our case, US imaging was sufficient to discover the testicular lesions that, indeed, were not detected by CT scan because the pelvis is not commonly investigated with this tool.

As previously discussed, the patient was found to have the bilateral testicular metastasis when he was 32 years old. According to the literature, the mean age of the patients with solid tumors metastatic to the testis is 60 years (range, 32–83 years). However, unlike metastases from the prostate are seen in the elderly, about one-third of patients with testicular metastasis from other cancer than prostatic one can present at a relatively younger age, as in the present case. Indeed, secondaries from the stomach and small bowel occur at a mean age of 31 years (31). Price & Mostofi (36) and Hanash *et al.* (37) reported 20–40% of cases occurring in patients less than 40 years old. Moreover, this is a hereditary case due to a germline RET mutation, and according to the phenomenon of the anticipation in genetic diseases (33), all clinical manifestations may anticipate with respect to sporadic cases.

Despite being young, the patient was affected by an advanced, diffuse metastatic MTC. In addition to cervical and mediastinal lymph nodes and lung metastases, the patient developed testicular metastasis via a probable hematogenous spread by arterial embolism. Several routes of tumor spread to the testes are described, such as retrograde venous or lymphatic extension and many others (23, 24, 31, 34), but they are less likely in the present case.

To the best of our knowledge, we reported the first case of asymptomatic and bilateral testicular metastases from MTC. Although exceptional, testis should be considered as a possible site of metastases in patients with diffuse metastatic MTC. In this setting, testicular US is helpful and sufficient to reveal an asymptomatic and often unnoticed secondary lesions to the testis, and it could be considered as an useful tool for the evaluation and follow-up of metastatic MTC.

## Declaration of interest

The authors declare that there is no conflict of interest that could be perceived as prejudicing the impartiality of this case report.

## Funding

This work did not receive any specific grant from any funding agency in the public, commercial or not-for-profit sector.

## Statement of ethics

The subject has given his written informed consent to publish the case, including publication of images.

## Author contribution statement

F O collected data, reviewed literature and wrote the manuscript. D C performed testicular ultrasound and reviewed the manuscript. F M performed surgical intervention. F B and G A performed histopathology analysis. A P and P F reviewed the manuscript and contributed to the discussion. P V, R E and E M critically revised the final manuscript.
